# The value of CT-based radiomics in predicting hemorrhagic transformation in acute ischemic stroke patients without recanalization therapy

**DOI:** 10.3389/fneur.2024.1255621

**Published:** 2024-02-01

**Authors:** Yin-hui Huang, Zhen-jie Chen, Ya-fang Chen, Chi Cai, You-yu Lin, Zhi-qiang Lin, Chun-nuan Chen, Mei-li Yang, Yuan-zhe Li, Yi Wang

**Affiliations:** ^1^Department of Neurology, Jinjiang Municipal Hospital (Shanghai Sixth People’s Hospital Fujian Campus), Quanzhou, China; ^2^Department of Neurology, Anxi County Hospital, Quanzhou, Fujian, China; ^3^Department of Neurology, The Second Affiliated Hospital of Fujian Medical University, Quanzhou, China; ^4^Department of CT/MRI, The Second Affiliated Hospital of Fujian Medical University, Quanzhou, China

**Keywords:** radiomics, hemorrhagic transformation, acute ischemic stroke, recanalization, AIS, ECASS II

## Abstract

**Objective:**

The aim of this study is to investigate the clinical value of radiomics based on non-enhanced head CT in the prediction of hemorrhage transformation in acute ischemic stroke (AIS).

**Materials and methods:**

A total of 140 patients diagnosed with AIS from January 2015 to August 2022 were enrolled. Radiomic features from infarcted areas on non-enhanced CT images were extracted using ITK-SNAP. The max-relevance and min-redundancy (mRMR) and the least absolute shrinkage and selection operator (LASSO) were used to select features. The radiomics signature was then constructed by multiple logistic regressions. The clinicoradiomics nomogram was constructed by combining radiomics signature and clinical characteristics. All predictive models were constructed in the training group, and these were verified in the validation group. All models were evaluated with the receiver operating characteristic (ROC) curve, calibration curve, and decision curve analysis (DCA).

**Results:**

Of the 140 patients, 59 experienced hemorrhagic transformation, while 81 remained stable. The radiomics signature was constructed by 10 radiomics features. The clinicoradiomics nomogram was constructed by combining radiomics signature and atrial fibrillation. The area under the ROC curve (AUCs) of the clinical model, radiomics signature, and clinicoradiomics nomogram for predicting hemorrhagic transformation in the training group were 0.64, 0.86, and 0.86, respectively. The AUCs of the clinical model, radiomics signature, and clinicoradiomics nomogram for predicting hemorrhagic transformation in the validation group were 0.63, 0.90, and 0.90, respectively. The DCA curves showed that the radiomics signature performed well as well as the clinicoradiomics nomogram. The DCA curve showed that the clinical application value of the radiomics signature is similar to that of the clinicoradiomics nomogram.

**Conclusion:**

The radiomics signature, constructed without incorporating clinical characteristics, can independently and effectively predict hemorrhagic transformation in AIS patients.

## Highlights

- The research object are these acute ischemic stroke patients who without recanalization therapy.

## Introduction

Acute ischemic stroke (AIS) is characterized by a high incidence, high fatality rate, and high disability rate, making it one of the leading causes of death worldwide (1). Hemorrhagic transformation (HT) is a common and serious complication of acute ischemic stroke, with reported incidences ranging from 10 to 40% in the literature (2). The European Cooperative Acute Stroke Study II (ECASS II) classifies HT into four distinct types: (a) hemorrhagic infarct type 1 (HI1), characterized as small spot hemorrhages along the infarct margin; (b) hemorrhagic infarct type 2 (HI2), defined by patchy or confluent spotting hemorrhages within the infarct area without significant mass effect; (c) parenchymal hematoma type 1 (PH1), involving hematomas less than 30% of the infarcted area with a mild mass effect; and (d) parenchymal hematoma type 2 (PH2), which includes hematomas exceeding 30% of the infarcted area and presenting a substantial mass effect (3). Notably, the PH2 type of HT has an alarmingly high in-hospital mortality rate of 52.3% (2). HT frequently leads to the deterioration of neurological functions, potentially resulting in life-threatening conditions (4), and significantly impacts patient prognosis (5).

Most hemorrhagic transformations are caused by the natural course of AIS, but they can also result from thrombolysis, interventional thrombectomy, and other recanalization therapies (6). Recanalization therapy such as thrombolysis and interventional thrombectomy is currently considered to be the most effective treatment for acute ischemic stroke, which has been proven to significantly improve the neurological impairment and prognosis of patients (7, 8). However, because of its narrow treatment time window, not many patients can receive recanalization therapy. Therefore, for the vast majority of patients who have not received recanalization treatment, it is more important to predict the occurrence of hemorrhagic transformation in the early stages.

Early identification of patients at high risk of hemorrhagic transformation will lead clinicians to reduce the dose of antithrombotic drugs, such as aspirin, adopt more neutral treatment options, and conduct more frequent clinical evaluations and monitoring (9). Furthermore, the re-examination time of cranial CT can be appropriately shortened and the frequency of cranial CT can be increased to detect the hemorrhagic transformation as soon as possible, which has guiding value for the subsequent adjustment of the treatment plan in clinical practice (10). For patients with a lower risk of hemorrhagic transformation, clinicians can take more active treatment plans to appropriately reduce the frequency of clinical monitoring and cranial CT examination so that patients can get more clinical benefits and reduce treatment costs. Therefore, the technical method that can accurately predict the hemorrhagic transformation in AIS patients is helpful for clinicians to make individual and accurate clinical treatment plans.

In recent years, radiologic features, such as the high-density sign of the middle cerebral artery, low density on CT, or abnormal diffusion-weighted imaging (DWI) signals on admission, and very low cerebral blood volume, have been considered predictive of hemorrhagic transformation. These may change the clinical management plan of patients and help clinicians in early prevention before hemorrhagic transformation occurs (2). However, the current research shows that it is not enough to rely on these radiologic features to predict hemorrhagic transformation, so it is necessary to explore a more accurate and objective model to predict the hemorrhagic transformation of AIS. It is also pointed out in the literature that the predictive model of hemorrhagic transformation also has the value of prognosis evaluation to a certain extent (11).

A variety of machine learning algorithms, combined with medical imaging, have been used in the diagnosis and prognosis of AIS. A multicenter study showed that the attention-gated U-Net deep learning algorithm with DWI and MRI perfusion as inputs could predict the final infarct volume, independent of the reperfusion state, and significantly overlap with the basic performance of the fluid-attenuated inversion recovery (FLAIR) sequence obtained 3–7 days after onset (12). Machine learning algorithms, including regularized logistic regression, linear support vector machines, and random forests, are superior to existing pre-treatment scoring methods in predicting the clinical outcome of patients with macrovascular occlusion undergoing thrombectomy. The AUC of the machine learning model is 0.85–0.86, while the AUC of the pre-treatment score is 0.71–0.77 (13). Previous studies have found that the hemorrhagic transformation after AIS was related to clinical factors such as hypertension, age, hyperglycemia, and stroke severity (14, 15). The use of a machine learning algorithm to combine medical image quantitative information with clinical information can effectively predict hemorrhagic transformation.

In this study, multiple machine learning models were constructed to predict hemorrhagic transformation after AIS based on the radiomics features of the infarcted area in CT plain scan images. Then the optimal machine learning model and clinical factors were combined to construct a nomogram for the risk assessment of hemorrhagic transformation in individualized AIS patients.

## Materials and methods

### Patients

The study was conducted following the Declaration of Helsinki and was approved by the ethics committees of participating hospitals, with the requirement for informed consent waived.

**Figure 1:**
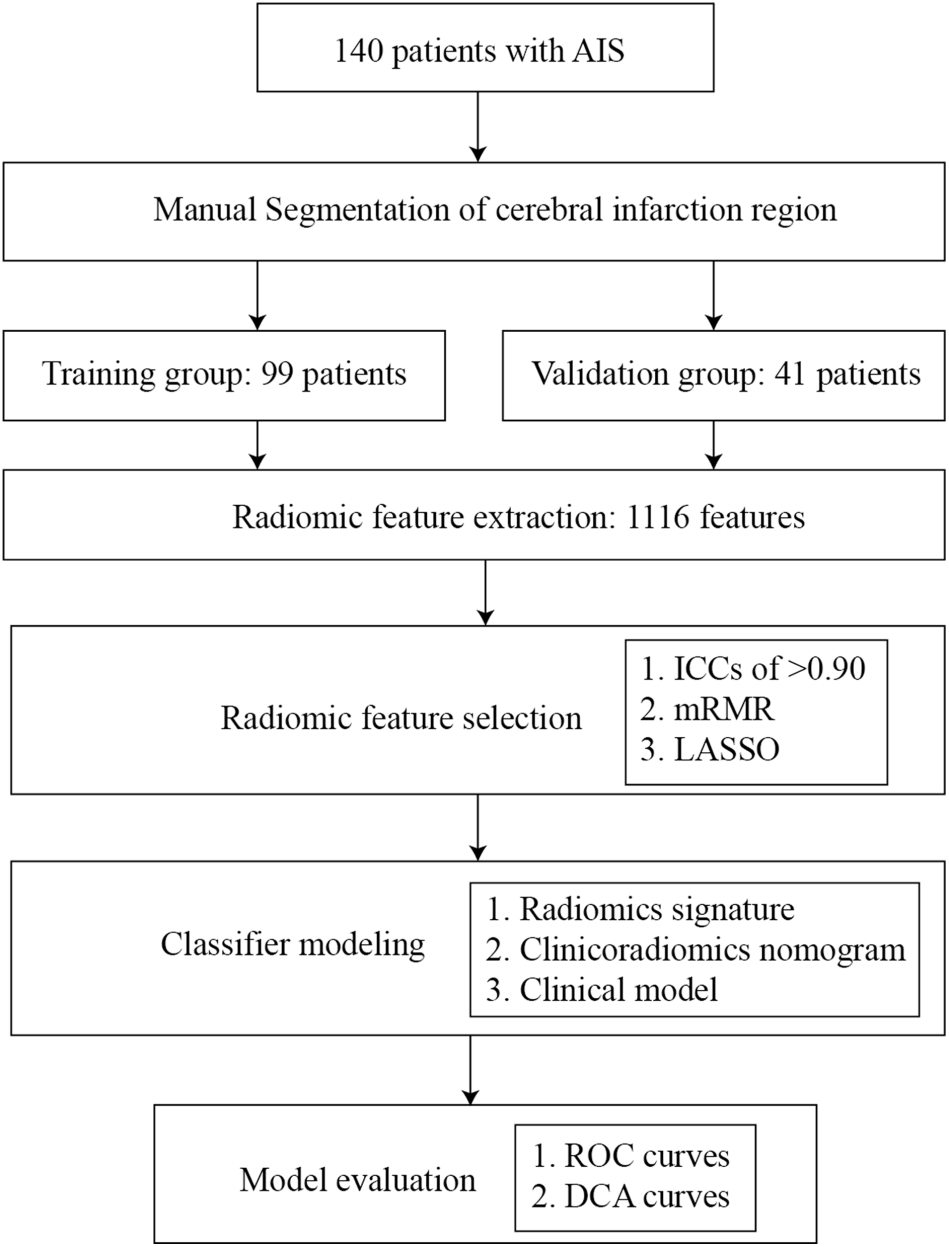
Diagram of the research design.

This retrospective, two-center study collected data on patients with AIS from January 2015 to August 2022. The diagnostic criteria for AIS and hemorrhagic transformation are based on the criteria established by the European Cooperative Acute Stroke Study (ECASS) (15). The inclusion criteria were as follows: (a) the time of onset was more than 1 day and less than 14 days; (b) responsible lesions could be seen in cranial CT; (c) patients did not undergo recanalization therapies such as intravenous thrombolysis or interventional thrombectomy due to their late arrival at the hospital, occurring more than 24 h after the onset of the stroke; and (d) re-examination of CT in 7 days. The exclusion criteria were as follows: (a) there is a history of cerebral hemorrhage, cerebrovascular malformation, brain tumor, brain trauma, or brain surgery; (b) participating in other studies and receiving additional drugs or treatment; (c) image artifacts or other factors affect image quality; and (d) AIS has occurred for more than 14 days. According to stratified sampling, the enrolled datasets were divided into a training group and a validation group at 7:3. The flow chart of the research design is displayed in Figure 1.

### Clinical characteristics

The following clinical characteristics were collected for patients with AIS: age, gender, previous vascular risk factors (such as hypertension, diabetes, hyperlipidemia, and smoking), atrial fibrillation, past stroke, transient ischemic attack (TIA), blood pressure, blood glucose, blood lipids, previously used drugs, the National Institute of Health stroke scale (NIHSS) score at admission, and a head CT plain scan before admission. Using relevant diagnostic tests, such as carotid ultrasound, and based on the outcomes of these tests, categorize the stroke subtypes as per the Trial of Org 10172 in Acute Stroke Treatment (TOAST) classification. A head CT plain scan was performed on the seventh day after treatment, and the CT was re-examined at any time when the symptoms of stroke worsened. According to the re-examination of the head CT, the patients were divided into a hemorrhagic transformation group and a non-hemorrhagic transformation group.

### CT data acquisition

All head CT plain scan images were obtained from six multi-slice CT scanners at the Department of CT/MRI, The Second Affiliated Hospital of Fujian Medical University (Brilliance 64 or Brilliance iCT, Incisive 60, Philips Healthcare; SOMATOM Definition DS CT, Siemens Healthcare; Optima CT520 or Optima CT620, GE Healthcare). The scanning parameters were as follows: tube voltage: 120 kVp; tube current: Optima CT520 226 mA, Brilliance 64,228 mA, Incisive 60,230 mA, automatic tube current modulation was using by other CT scanner; field of view (FOV): FOV was adapted to the size of the patient; matrix size: 512 × 512; section thickness: 5 mm; section interval: 5 mmNAC regimen.

### Cerebral infarction region segmentation

**Figure 2:**
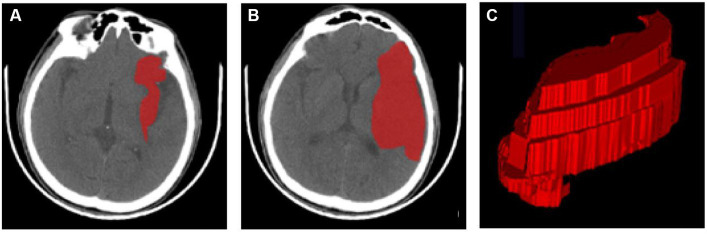
ROI segmentation using ITK-SNAP. **(A,B)** ROIs of the infarct area at different axial levels. **(C)** A 3D-ROI of the infarcted region. 3D three-dimensional, CT computed tomography, ROI region of interest.

ITK-SNAP (version 3.8.0) was used for the manual segmentation of the region of interest (ROI) (1). The segmentation was carried out along the edge of the infarcted area on each slicer of the CT image (Figure 2). Reader 1, who was blinded to the patient information, segmented all ROIs. Then, all ROIs were segmented again by the same radiologist after 1 month to evaluate the intra-observer agreement. Another neurologist segmented all cases over the same period to evaluate the inter-observer agreement. The intra-class correlation coefficient (ICC) was applied to assess the reproducibility of radiomic feature extraction, and a two-way mixed-effects model was used to calculate the ICC [17]. In this study, radiomic features of ICC > 0.90 were selected and considered a mark of satisfactory intra- and inter-observer agreement.

### Radiomic feature extraction

A total of 1,316 radiomic features were extracted from the cerebral infarction region using the AK application belonging to the PHIgo workstation (Version 1.5.0 R), which can be roughly divided into the following categories: first-order statistics, shape-based, gray level co-occurrence matrix (GLCM), gray level size zone matrix (GLSZM), gray level run length matrix (GLRLM), neighboring gray tone difference matrix (NGTDM), and gray level dependence matrix (GLDM).

### Classifier modeling

Max-relevance and min-redundancy (mRMR) and the least absolute shrinkage and selection operator (LASSO) were successively used for radiomic feature selection. Based on the heuristic scoring criteria, radiomic features with ICCs >0.90 were ranked by the mRMR method according to their relevance-redundancy index, and the top 10 features with high relevance were retained. The optimized subset of features was then selected from the retained features by the LASSO classifier. The radiomics signature was then constructed by multiple logistic regressions based on the selected radiomic features. Rad-score was calculated by summing the selected features weighted by their coefficients.

To assess the disparities in clinical characteristics between the hemorrhagic transformation group and the non-hemorrhagic transformation group, we conducted both univariate and multivariate analyses. Subsequently, we identified independently significant clinical characteristics and utilized them to develop a predictive model for hemorrhagic transformation using logistic regression. Additionally, we constructed a comprehensive clinicoradiomics nomogram that integrated the radiomic features derived from the radiomics signature with the clinical characteristics identified from the logistic regression model.

### Statistical analysis

The clinical characteristics between the hemorrhagic transformation group and the non-hemorrhagic transformation group were evaluated using the two independent sample t-test or Mann–Whitney U-test accordingly. The predictive performance of machine learning models was evaluated using the receiver operating characteristic (ROC) curve. Differences in area under the ROC curves (AUCs) between the models were evaluated using Delong’s test. In addition, only the machine learning model with the highest AUC for each sequence was used for Delong’s test. *p* < 0.05 were considered significant. The data were analyzed using SPSS 25 (version 25), R software (version 4.0.4), and MATLAB (version 2022a).

## Results

### Demographics

**Figure 3:**
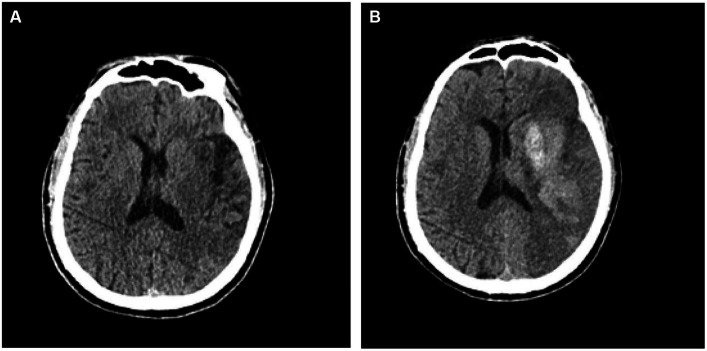
CT images depicting patients with acute ischemic stroke prior to **(A)** and subsequent to **(B)** hemorrhagic transformation.

A total of 140 patients with AIS were selected for this study. All patients were assigned randomly to the training group (*n* = 99) or the validation group (*n* = 41). In this cohort of AIS patients, 59 cases were identified as having undergone hemorrhagic transformation, while 81 cases remained stable. Within the hemorrhagic transformation group, 21 cases were classified as HI1, 15 cases as HI2, 11 cases as PH1, and 12 cases as PH2. The hemorrhagic transformation of AIS is displayed in Figure 3. Of the 140 patients, 86 were men and 54 were women. The mean age of the patients was 65.2 ± 12.2 years, range 39–93 years. In patients with hemorrhagic transformation and those without, significant differences were observed in fasting blood glucose (FBG), serum cholesterol, and NIHSS scores, as well as the presence of atrial fibrillation (*p* < 0.05). However, there were no significant differences between these two groups in terms of age, blood pressure upon admission, previous medications, diabetes, high cholesterol, hypertension, sex, smoking habits, history of stroke, or transient ischemic attack (*p* > 0.05). Table 1 displays the clinical characteristics of the subjects in both the training and validation groups.Radiomic signature construction.

**Table 1:** Clinical characteristics of patients.

Clinical factors	Entire group (*n* = 140)	Training group (*n* = 99)	Validation group (*n* = 41)	*p*-value
Age, years	65.7 (12.1)	66.2 (11.8)	64.6 (12.7)	0.513
Blood pressure on admission	146.0 (24.8)	146.6 (25.8)	146.3 (23.0)	0.468
FBG	7.0 (2.5)	7.2 (2.5)	6.6 (2.4)	0.001
Serum cholesterol	4.6 (1.5)	4.5 (1.4)	4.7 (1.6)	0.018
NIHSS	9.7 (7.7)	9.8 (7.5)	9.7 (6.8)	0.851
Atrial fibrillation	27 (19.2)	19 (19.2)	8 (19.5)	< 0.001
Diabetes	30 (21.4)	28 (28.3)	2 (4.9)	0.571
High cholesterol	1 (0.7)	0 (0.0)	1 (2.4)	1.000
Hypertension	65 (46.4)	46 (46.5)	19 (46.3)	0.114
Sex (male)	86 (61.4)	61 (61.6)	25 (61.0)	0.430
Smoking	27 (19.3)	20 (20.2)	7 (17.1)	0.550
Stroke history	25 (17.9)	16 (16.2)	9 (22.0)	0.122
Volumetry in cm^3^	18.86 (9.37)	18.63 (9.54)	18.91 (9.15)	0.719
TOAST categorization				
Large-artery atherosclerosis	78 (55.71)	55 (55.56)	23 (56.10)	0.953
Cardioembolism	26 (18.57)	18 (18.18)	8 (19.51)	0.854
Small-vessel occlusion	23 (16.43)	16 (16.16)	7 (17.07)	0.895
Stroke of other determined etiology	8 (5.71)	6 (6.06)	2 (4.88)	0.784
Stroke of undetermined etiology	5 (3.60)	4 (4.04)	1 (2.44)	0.642

For continuous variables, the data are expressed as mean (SD). For categorical variables, they are presented as count (percentage). NIHSS, National Institute of Health stroke scale; TOAST, Trial of Org 10,172 in acute stroke treatment; SD, standard deviation; FBG, fasting blood glucose.

**Figure 4:**
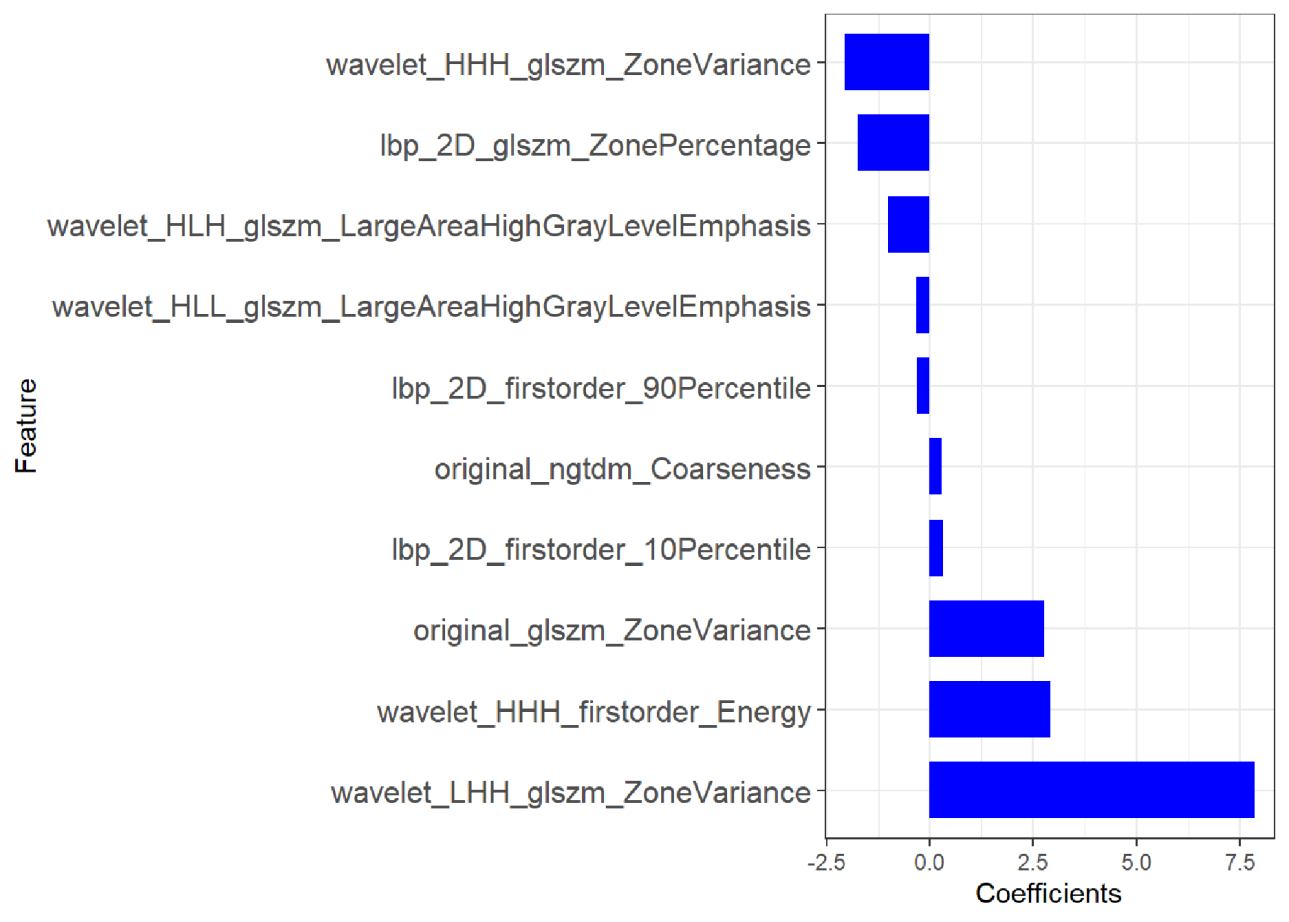
Most predictive subset of radiomics feature was chosen, along with the corresponding coefficients.

Of the 1,116 radiomic features, 923 features with intra- and inter-observer ICCs >0.80 were retained. mRMR eliminates redundant and irrelevant features for stable radiomic features, and 20 features were retained. Based on the training group, the 10 most significant features were selected by LASSO to build the radiomic signature. The 10 most significant feature names and corresponding coefficients are displayed in Figure 4.

### Diagnostic ability of radiomic signature

**Figure 5:**
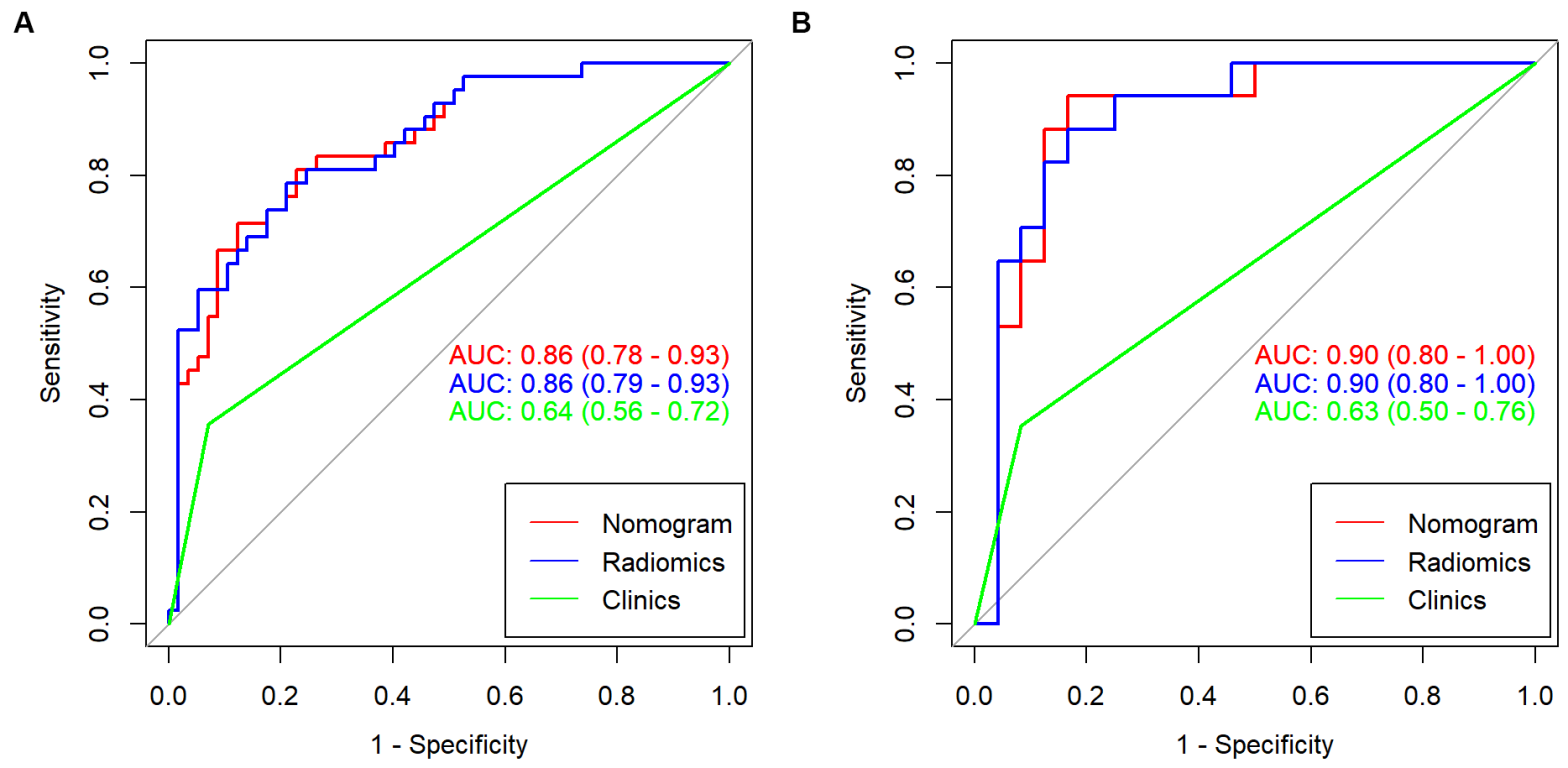
ROC curves of radiomics signature in the training group **(A)** and validation group **(B)**.

In the training group, the AUC value of the radiomic signature was 0.86 (95% CI: 0.79–0.93). In the validation group, the AUC value of the radiomic signature was 0.90 (95% CI 0.80–1.00). Further information can be found in Table 1 and Figure 5.

### The diagnostic ability of nomogram

**Figure 6:**
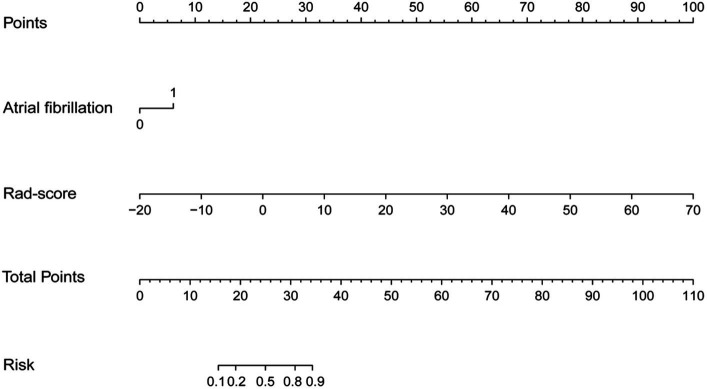
Clinicoradiomics nomogram.

**Figure 7:**
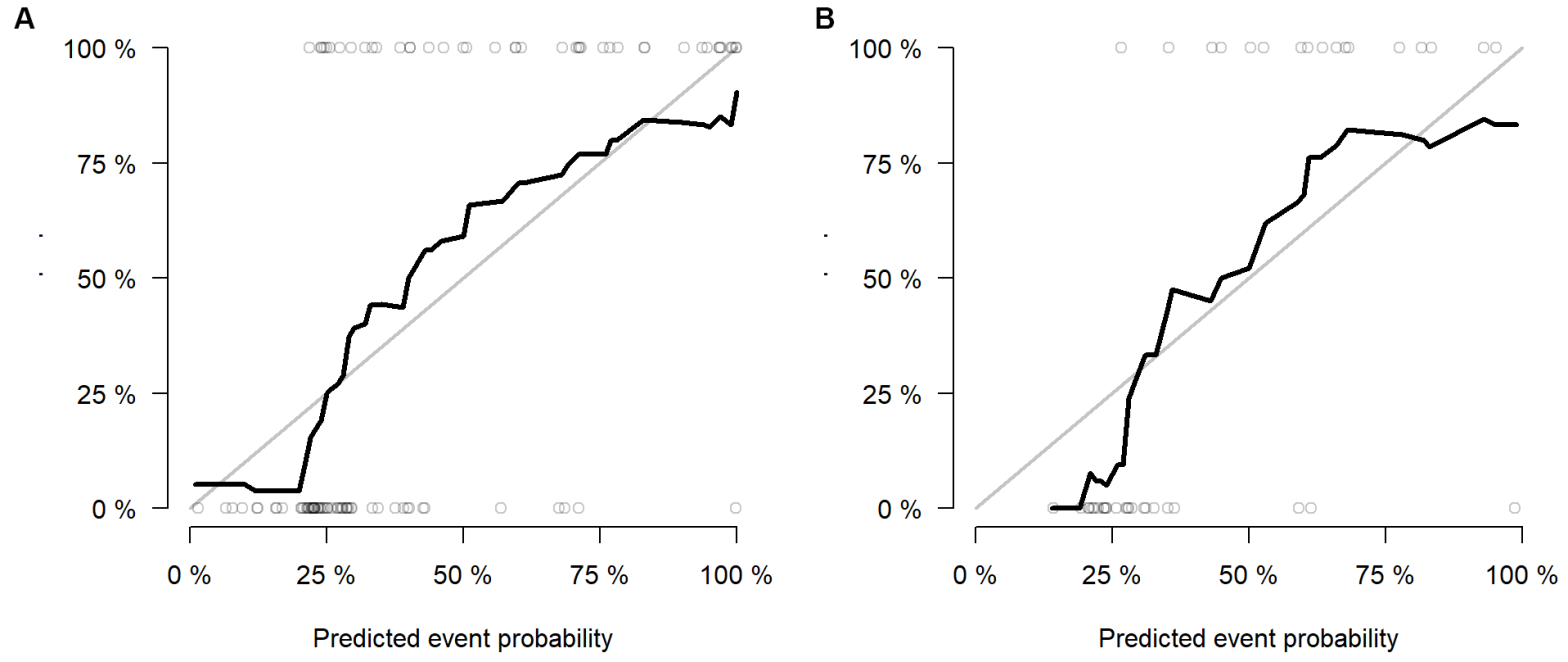
Calibration curves of radiomics signature in the training group **(A)** and validation group **(B)**.

**Figure 8:**
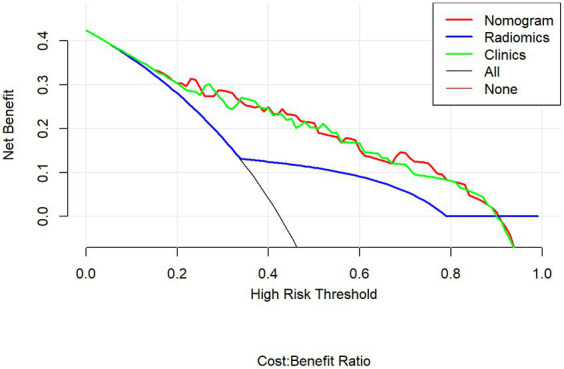
Decision curve analysis curve.

Multivariable logistic regression analysis revealed that the Rad-score and atrial fibrillation were significant independent factors for AIS with hemorrhage transformation (Figure 6). The clinical model was also constructed using multivariate logistic regression analysis. For the clinical model, the AUCs with the training group and the validation group were 0.64 (95% CI 0.56–0.72) and 0.63 (95% CI 0.50–0.76), respectively. For the clinicoradiomics nomogram, the AUCs with the training group and the validation group were 0.86 (95% CI 0.78–0.93) and 0.90 (95% CI 0.80–1.00), respectively (Table 2; Figure 5). The calibration curve indicated a strong level of agreement between the actual and predicted diagnoses in both groups (Figure 7). The results of the Hosmer–Lemeshow test were not statistically significant, with a *p*-value of 0.33 for the training group and 0.15 for the validation group. Decision curve analysis (DCA) curves showed that the radiomics signature and clinicoradiomics nomogram were significantly better than the clinical model in predicting AIS with hemorrhage transformation (Figure 8).

**Table 2:** Diagnostic performance of the radiomic signature in training and validation groups.

Models	Groups	AUC	95% CI	Accuracy	Sensitivity	Specificity
Radiomics signature	Training group	0.86	0.79–0.93	0.79	0.79	0.79
	Validation group	0.90	0.80–1.00	0.83	0.88	0.79
Clinicoradiomics nomogram	Training group	0.86	0.78–0.93	0.81	0.71	0.81
	Validation group	0.90	0.80–1.00	0.88	0.80	0.94
Clinical model	Training group	0.64	0.56–0.72	0.69	0.36	0.79
	Validation group	0.63	0.50–0.76	0.68	0.35	0.75

AUC, area under the curve; CI confidence interval.

## Discussion

In recent years, imaging has played an important role in the diagnosis, treatment, and prognosis of cerebrovascular diseases. Hemorrhagic transformation is closely related to the poor prognosis of patients with AIS, and it is also an important reference index for clinical treatment (16). Previous studies have established models based on imaging features to predict the risk of hemorrhagic transformation in patients with AIS after thrombolysis or interventional thrombectomy and showed good predictive efficiency (17–19). However, as far as we are aware, there are few studies on predicting the risk of hemorrhagic transformation in patients who do not receive recanalization therapy, such as thrombolysis or interventional thrombectomy, which account for the majority of AIS cases, based on imaging characteristics.

Similar to previous studies, we also found that atrial fibrillation was an independent risk factor for hemorrhagic transformation (20, 21). As is widely known, the cardiogenic emboli formed by atrial fibrillation are usually large and can easily block large arteries such as the internal carotid artery and middle cerebral artery, leading to large-area cerebral infarctions. A large number of studies show that large-area cerebral infarction is more likely to occur than hemorrhagic transformation. The mechanism may be that when a large cerebral infarction occurs, brain tissue ischemia and hypoxia lead to severe cytotoxic edema, which exacerbates the disruption of the blood–brain barrier, thus leading to the occurrence of hemorrhagic transformation (17, 22). However, the results of this study showed that the radiomics signature was less disturbed by clinical factors, and the predictive efficiency was significantly better than the traditional clinical model.

In this study, the clinical model, radiomics signature, and clinicoradiomics nomogram were constructed to predict hemorrhagic transformation in AIS patients who did not receive thrombolysis or interventional thrombectomy. The results showed that the head CT-based radiomics signature and clinicoradiomics nomogram both had good predictive performance, and the validation accuracy was 0.83 and 0.88, respectively. However, according to the DCA curve, the clinical application value of the clinicoradiomics nomogram is consistent with that of the radiomics signature. In the absence of detailed clinical data and hematological indicators at the initial stage of the disease, the risk of hemorrhagic transformation can be predicted only according to the radiomic features based on CT images, which provides an important reference for clinicians to early evaluate the risk of hemorrhagic transformation and make a preliminary treatment plan. Early and accurate identification of high-risk AIS patients with hemorrhagic transformation can enable clinicians to carry out more stringent clinical monitoring and head CT re-examination in the early stages to prevent hemorrhagic transformation and improve the prognosis of patients (9). In addition, according to the predicted results of the model, the risk of hemorrhagic transformation in patients with AIS can be stratified to help clinicians make individual and accurate clinical treatment plans.

A limitation of this study is the small number of patients and its retrospective nature. However, to validate these preliminary findings, it is imperative to conduct further prospective investigations. The development of automatic segmentation for cerebral hemorrhage is urgently needed. This study only focused on AIS patients with hemorrhagic transformation, which results in selection bias and lower power.

In short, early prediction of hemorrhagic transformation is very important, and the radiomics signature, which was constructed without clinical characteristics, can independently predict hemorrhagic transformation in AIS effectively. This radiomics signature may change the clinical management plan of patients, improve clinical treatment decisions, and benefit more patients.

## Data availability statement

The original contributions presented in the study are included in the article/supplementary material, further inquiries can be directed to the corresponding authors.

## Ethics statement

The study was conducted following the Declaration of Helsinki and was approved by the ethics committee of participating hospitals, with the requirement for informed consent waived.

## Author contributions

Y-hH: Data curation, Formal analysis, Investigation Writing – review & editing. Z-jC: Data curation, Formal analysis, Investigation, Writing – review & editing. Y-fC: Data curation, Formal analysis, Investigation, Writing – review & editing. CC: Data curation. Y-yL: Formal analysis. Z-qL: Investigation. C-nC: Formal analysis. M-lY: Methodology, Project administration, Software, Writing – review & editing. Y-zL: Data curation, Formal analysis, Funding acquisition, Writing – review & editing. YW: Data curation, Formal analysis, Writing – review & editing.
